# Lithium Accumulation in *Salvinia natans* Free-Floating Aquatic Plant

**DOI:** 10.3390/ma15207243

**Published:** 2022-10-17

**Authors:** Anamaria Iulia Török, Ana Moldovan, Eniko Kovacs, Oana Cadar, Anca Becze, Erika Andrea Levei, Emilia Neag

**Affiliations:** 1 Research Institute for Analytical Instrumentation Subsidiary, National Institute for Research and Development for Optoelectronics INOE 2000, 67 Donath Street, 400293 Cluj-Napoca, Romania; 2 Faculty of Horticulture, University of Agricultural Sciences and Veterinary Medicine, 3–5 Manastur Street, 400372 Cluj-Napoca, Romania

**Keywords:** *Salvinia natans*, lithium, bioconcentration factor, photosynthetic pigments

## Abstract

The new context of the intensive use of lithium-based batteries led to increased production of Li and Li-containing wastes. All these activities are potential sources of environmental pollution with Li. However, the negative impact of Li on ecosystems, its specific role in the plants’ development, uptake mechanism, and response to the induced stress are not fully understood. In this sense, the Li uptake and changes induced by Li exposure in the major and trace element contents, photosynthetic pigments, antioxidant activity, and elemental composition of *Salvinia natans* were also investigated. The results showed that *Salvinia natans* grown in Li-enriched nutrient solutions accumulated much higher Li contents than those grown in spring waters with a low Li content. However, the Li bioaccumulation factor in *Salvinia natans* grown in Li-enriched nutrient solutions was lower (13.3–29.5) than in spring waters (13.0–42.2). The plants exposed to high Li contents showed a decrease in their K and photosynthetic pigments content, while their total antioxidant activity did not change substantially.

## 1. Introduction

There is a vast number of both terrestrial and aquatic plant species that can uptake pollutants from water, soil, and even from the air [1,2,3,4]. The accumulation of metals in aquatic plants depends on the concentration of the metals in water and plant species and the exposure time. Several processes, such as element mobilization, root uptake, xylem loading, root-to-shoot transport, cellular compartmentation, and sequestration, are involved in the accumulation of metals in plants [5]. However, the specific mechanism of metal accumulation in plants is not entirely understood. Metals in ionic form can attach to the surface of plants very quickly, being integrated into the plant’s membrane surface or fixed on the sites used for nutrient uptake [6]. The bioavailability of metals to plants and the changes induced in the plants by different metals as well as their defense mechanisms were previously studied [5]. Chlorophyll is responsible for collecting solar energy during photosynthesis, while carotenoids eliminate the reactive oxygen species in the damaged tissues [7]. Changes in the photosynthetic pigments indicate the presence of stress factors, such as toxic elements. Exposure to toxic elements also stimulates the formation of reactive oxygen species and other highly reactive compounds, damaging the plant’s lipids, proteins, and nucleic acids [8]. By simultaneous exposure to various elements, the plants face multiple metal stresses, as well as competition between nutrients (K, Ca, Na, Mg), essential (Cu, Zn) and toxic (Cd) elements. High concentrations of nutrients and essential elements may also activate the protective mechanism of plants [9,10].

Aquatic macrophyte species, grouped in emergent, submerged, and free-floating plants, can accumulate a wide range of pollutants, such as toxic metals, pesticides, and radionuclides from waters and wetlands [11,12]. The pollutant’s type and location determine the processes of its removal and breakdown by macrophytes [13]. Free-floating aquatic plants are ideal for phytoextraction and rhizofiltration, as they are easy to grow and harvest [9]. The Salviniaceae aquatic species have a remarkable ability to uptake, tolerate and bioaccumulate toxic inorganic pollutants [14,15,16,17], phosphates [18], nitrogen [19], arsenic [20], and amino groups from aqueous solutions [21]. *Salvinia natans* (L.) All. (*S. natans*) is a free-floating fern from the Salviniaceae family and has a high tolerance to a wide range of pollutants and a rapid growth rate [22]. It is native to Europe and widely spread within temperate, tropical, and subtropical regions [16,23,24]. *S. natans* has two floating leaves and a submerged leaf, which functions as a root, assimilating nutrients and pollutants [25]. The leaves are predominantly composed and covered by eggbeater-shaped hairs, making them hydrophilic. In order to determine the accumulation potential of *S. natans*, its tolerance to various heavy metals and trace elements was studied [16,17,26,27,28].

Among the trace elements, lithium (Li) is a critical element used in energy storage, alloys for military and aerospace applications, lubricating greases, glass and ceramics, antidepressant drugs, and hydrogen storage [29]. The use of Li to produce Li-ion batteries steeply increased in the last decade, triggering an increase in Li production and generating Li-containing wastes. As the large-scale recycling of batteries and other Li-containing wastes is still challenging, the contamination of the environment with Li has an increasing trend [30,31]. Because of the small ion radius, Li is a biotic element that is highly soluble in aqueous solutions, frequently forming free cations with a low charge [32,33]. The concentrations of Li vary depending on the nature of the water (i.e., surface water, groundwater, seawater), being a scarce element in natural water [31,33]. The Li concentration varies widely in surface waters. For example, the average Li value was around 40 μg/ L in the USA [34], while it varied from 0.5 to 15.0 μg/L in the Republic of Macedonia [35] and between 20 and 91 μg/L in Ireland [36]. Except for the Austrian, Argentinean, and Chilean regions, where the Li concentrations were reported to exceed 1000 μg/L, the concentration of Li in groundwater ranged from 0.05 to 150 μg/L [37]. In seawater, Li concentrations between 170–190 μg/L were found [38]. In Romania, there is limited information regarding Li’s concentration in water. Dippong et al. [39] reported average concentrations of Li between 1.20 and 114 μg/L in Romanian bottled water brands, while Zsigmond et al. [40] found high Li concentrations in most of the studied Transylvanian mineral waters. Torok et al. [41] reported Li concentrations of 1.40–12.20 µg/L in groundwaters from the karst areas in Romania.

Li is not considered an essential element in plant growth and development, yet some studies revealed its beneficial role in plants. Due to the physicochemical similarities between Li, Na, K, and Ca, these elements can be easily uptaken by the plant’s roots through the same transport systems [42]. Li bioavailability, phytotoxicity, and its interaction with other elements, as well as the uptake mechanism and the specific role in plant species development, are not fully elucidated [42,43]. Moreover, the understanding of the dose–response relationship and short- and long-term influence on plant physiology is not currently elucidated.

The present study aimed to evaluate the Li uptake by *S. natans* from mono- and multiple-element solutions, as well as from natural spring waters, and to observe the Li-induced changes in the plant’s major and trace element contents. At the same time, the plant’s vital signs—as a response to the induced stress—were monitored through the photosynthetic pigments content and total antioxidant activity.

## 2. Materials and Methods

### 2.1. Experimental Design

*S. natans* aquatic plants were obtained from a local aquarium store in Cluj-Napoca, Romania. Prior to the experiments, plant species were grown for 30–40 days under laboratory conditions in the Hoagland nutrient solution containing 1.25 mM KNO_3_, 1.25 mM Ca(NO_3_)_2_, 0.5 mM MgSO_4_, 0.25 mM KH_2_PO_4_, 10 μM FeEDTA, 11.6 μM H_3_BO_3_, 4.5 μM MnCl_2_ ·4H_2_O, 0.19 μM ZnSO_4_ ·7H_2_O, 0.12 μM Na_2_MoO_4_· 2H_2_O, and 0.08 μM CuSO_4_·5H_2_O [44]. Metal accumulation experiments were carried out in the Hoagland solution enriched with concentrations of 10 mg-Li/L, 20 mg-Li/L, 50 mg-Li/L, and 20 mg-Li/L, 20 mg-Cu/L, 20 mg-Zn/L, and 20 mg-Cd/L by dissolving LiSO_4_·H_2_O, CuSO_4_·5H_2_O, ZnSO_4_·7H_2_O, and Cd(NO_3_)_2_·5H_2_O, as well as in spring waters with different Li concentrations. Spring water samples were collected in November 2021 from the Banpotoc (W1, Hunedoara county, Romania), Borcut (W2, Maramures county, Romania), and Botiza villages (W3, Maramures county, Romania). The major and trace elements in the spring waters are presented in Table 1.

Amounts of 2.5 g (fresh weight, FW) of *S. natans* were placed in 250 mL of enriched Hoagland solution or spring waters. For the control experiments, the Hoagland nutrient solution was used. Plants were left to grow at room temperature (20–23 °C) under natural light conditions for 7 days and then were harvested and analyzed. The *S. natans*’ life span is approximately 130–200 days and includes gametophytic and sporophytic phases [23,45]. Generally, sporophyte development requires about 170 days, while gametophyte development requires approximately 35 days [46]. Previous investigations showed that a 6–7 day growth period is enough for the aquatic macrophytes to uptake and accumulate metals, such as Cu, Zn, and Cd, from multi-metal solutions and to develop responses to the induced stress [9,10,47]. All experiments were performed in triplicate (*n* = 3). The samples of *S. natans* after Li exposure were denoted as follows: (i) control *S. natans*—SC; (ii) *S. natans* exposed to 10 mg-Li/L—S10; (iii) *S. natans* exposed to 20 mg-Li/L—S20; (iv) *S. natans* exposed to 50 mg-Li/L—S50; (v) *S. natans* exposed to 20 mg-Li/L, 20 mg-Cu/L, 20 mg-Zn/L, and 20 mg-Cd/L concentrations—SM20; (vi) *S. natans* exposed to spring water, SW1–SW3.

### 2.2. Element Analysis

The plant samples were washed with distilled water, oven-dried at 65 °C, ground using an agate mortar, and passed through a 200-µm mesh sieve to obtain a homogenized powder. An amount of 200 mg of plant material was digested with 5 mL of 65% HNO_3_ and 2 mL of 30% H_2_O_2_ (Merck, Darmstadt, Germany). The digested samples were diluted with ultrapure water to a final volume of 25 mL. The measured metal concentrations were expressed as mg/kg dry weight (DW). The Na, Mg, K, Ca, and Fe concentrations were measured using an Optima 5300 DV inductively coupled plasma–atomic emission spectrometer (ICP–OES, Perkin Elmer, Waltham, MA, USA), while the Li, Mn, Fe, Ni, Cu, Zn, Rb, Sr, Cd, and Ba concentrations were determined using an ELAN DRC II inductively coupled plasma–mass spectrometer (ICP–MS, Perkin Elmer, Waltham, MA, USA). ICP multi-element standard solution IV, 1000 mg/L (Merck, Darmstadt, Germany), and multi-element Calibration Standard 3 (Perkin Elmer Pure Plus, Waltham, MA, USA) were used for the calibration, while the measurement accuracy was tested using the standard reference material, 1643f NIST–Trace elements in water (National Institute of Standards and Technology, Gaithersburg, MD, USA) and NIM–GWB 10019 Apple–Trace elements (Institute of Geophysical and Geochemical Exploration, Langfang City, China) for plants. The average recoveries ranged between 93–104%. Ultrapure water (Elga Veolia, High Wycombe, UK) was used for dilutions and the preparation of the standard solutions. The N, C, H and S contents were determined using a Flash 2000 CHNS/O analyzer (Thermo Fisher Scientific, Waltham, MA, USA).

### 2.3. Photosynthetic Pigments

The photosynthetic pigments (chlorophyll *a*—*C_a_*, chlorophyll *b*—*C_b_*, and carotenoids—*C_x+c_*) were measured in an extract of 0.5 g of *S. natans* fresh weight (FW) in 5 mL of methanol. The plant–methanol mixture was vortexed thoroughly for 1 min and incubated at 70 °C for 3 min. The extracts were centrifuged, and the absorbance of the supernatant was measured at 665, 652, and 470 nm using a Nanodrop One Analyzer (Thermo Fisher Scientific, Waltham, MA, USA). The contents of the *C_a_*, *C_b_*, and *C_x+c_* pigments were calculated according to Lichtenthaler (1987), and the results were expressed as µg/g (FW) [48].

### 2.4. Antioxidant Content

An amount of 5 g of fresh *S. natans* was extracted using 20 mL of methanol. The samples were vortexed for 1 min and then centrifuged at 3500 rpm for 10 min. The supernatant was analyzed using Photochem (AnalytikJena, Jena, Germany). Trolox (ACL kit, AnalytikJena, Jena, Germany) was used as a standard for the quantitative assessment of antioxidant activity. The calibration curve was made using a range of pathlengths from 0.5 to 3 nm. The samples were diluted with methanol at 1:20 in order to be in the concentration range of the calibration curve.

### 2.5. FT–IR Analysis

The FT–IR spectra were recorded using a Spectrum BX II (Perkin Elmer, Waltham, MA, USA) Fourier–transform infrared spectrometer on pellets containing 1% (*w*/*w*) dried and powdered samples in KBr.

### 2.6. Bioaccumulation Factor

The metal bioaccumulation factor (BCF) was calculated according to Equation (1) [49,50].

BCF = CP/CW(1)

where, CP is the Li concentration in the plant (mg/kg) and CW is the initial Li concentration in the Hoagland solutions enriched with Li or in the spring waters (mg/L).

### 2.7. Data Analysis

The OriginLab (version 2020b) software (Northampton, MA, USA) was used for data analysis and the plotting of the charts. The differences between the studied parameters were tested by comparing the averages of the three replicates using Tukey’s test for a significance level of *p* = 0.05 using the OriginPro (version 2020b) software (OriginLab Corporation, Northampton, MA, USA).

## 3. Results and Discussion

### 3.1. Li Uptake by S. natans

**Figure 1:**
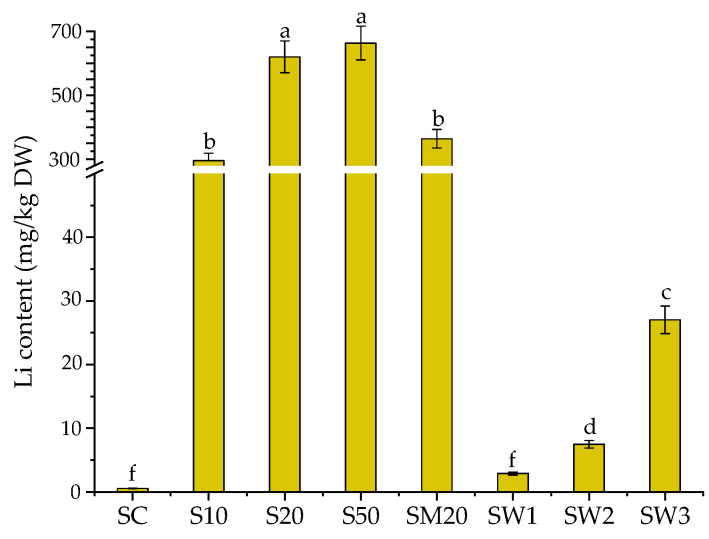
The Li content in *S. natans* grown in control (SC), Hoagland solution enriched with 10 mg-Li/L (S10), 20 mg-Li/L (S20), 50 mg-Li/L (S50), 20 mg-Li/L, 20 mg-Cu/L, 20 mg-Zn/L, and 20 mg-Cd/L (SM20), and Banpotoc (SW1), Borcut (SW2) and Botiza (SW3) spring waters. Li contents marked with different letters are significantly different at the *p* < 0.05 significance level.

*S. natans* showed an excellent ability to accumulate Li from the enriched Hoagland solutions. The Li content in *S. natans,* grown in the control solution, was 0.57 mg/kg DW and increased considerably in the Li-enriched Hoagland solutions. As shown in Figure 1, the Li uptake in the S10, S20, and S50 plants increased 500–1000 fold with the increased Li concentration in the test solution.

A steep increase in the Li content (295 mg/kg DW) was observed in the *S. natans* grown in the solution containing 10 mg-Li/L. This increase was also observed for the plants grown in the enriched Hoagland solution containing 20 mg-Li/L, with Li (621 mg/kg DW) being twice as high as those grown in S10. However, for the *S. natans* grown in 50 mg-Li/L solutions, no further increase in the Li content (664 mg/kg DW) was observed. These results indicated that, up to a concentration of 20 mg-Li/L in the growth medium, the accumulated Li in *S. natans* increased proportionally, but at higher Li concentrations, the accumulated Li remained more or less constant. This could be a consequence of the growth-stimulating effect of low Li concentrations and the toxic effects of high Li contents. Hawrylak-Nowak reported that 50 mg-Li/L (as the mono-element) in the growing medium was toxic for plants and that the toxicity symptoms, as well as the amount of accumulated Li, were species-specific [51,52]. The growth-stimulating effects of low Li concentrations were also reported for maize (5 mg-Li/L) [53] and lettuce (up to 14 mg-Li/L) [42]. Previous studies showed that exposure to the 50 mg-Li/L solutions resulted in the accumulation of 695 mg/kg DW for maize, 3292 mg/kg DW for sunflower, and 1607 and 1544 mg/kg DWs for lettuce [51,52].

The Li content in *S. natans,* grown in SM20, was considerably higher than in the control sample. However, it was almost half lower (364 mg/kg DW) than those grown in S20 (621 mg/kg DW), indicating a possible competition of Li with Cu, Zn and Cd for uptake [47,54]. The Li content in *S. natans* exposed to spring waters was lower (2.90 to 27.1 mg/kg DW) than in those exposed to the Li-enriched growing solution. The low uptake could be explained by the low Li concentration of spring waters. Previous studies have reported that *S. natans* exhibited a high ability to accumulate Cu and Cd from multi-element solutions [46,53].

### 3.2. Major and Trace Element Contents in S. natans Exposed to Li

**Figure 2:**
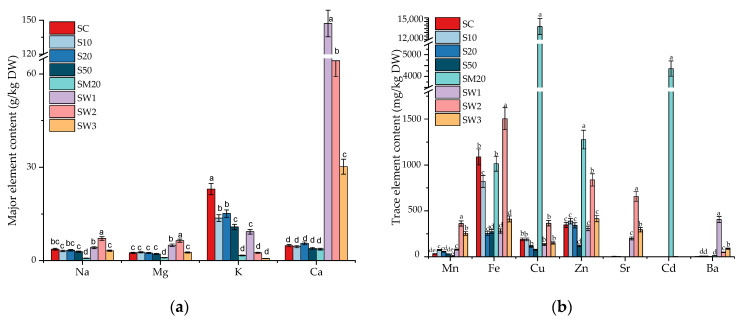
(**a**) Major and (**b**) trace element contents in *S. natans* grown in control (SC), Hoagland solution enriched with 10 mg-Li/L (S10), 20 mg-Li/L (S20), 50 mg-Li/L (S50), 20 mg-Li/L, 20 mg-Cu/L, 20 mg-Zn/L, and 20 mg-Cd/L (SM20), and Banpotoc (SW1), Borcut (SW2) and Botiza (SW3) spring waters. Element contents marked with different letters are significantly different at the *p* < 0.05 significance level.

Generally, the macro- and micro-element contents in aquatic plants depend on the chemical composition of the aquatic environment, the element of interest, and the plant species [55]. The major and trace element contents in the *S. natans* plants are presented in Figure 2. By simultaneous exposure of *S. natans* to Li, Cu, Zn, and Cd, the accumulation of all major elements decreased (Figure 2a), with the most drastic drop being observed for K (from 23 g/kg DW for the control to 1.64 g/kg DW for the SM20). This fact suggests that K competes with other elements for uptake and that the uptake of Li takes place through K transporters.

Compared to the control experiment, *S. natans* exposed to the W1–W3 springs showed several changes in the major and trace element contents. As the spring waters contain 30-, 13-, and 6-fold higher Ca concentrations than in the control sample, Ca was the highest accumulated element. The highest Ca content was measured in SW1 (147 g/kg DW), while the lowest was measured in SW3 (30.2 g/kg DW). The K content in the SW1–SW3 exhibited a decreasing tendency, probably due to the inhibition of the uptake that occurred during exposure to the spring waters. It was previously reported that Ca can inhibit the absorption of Li and normalize the K uptake under Li stress [32]. This fact was also observed in the current study, as the high Ca content in *S. natans* grown in SW1 led to a low Li content compared to those grown in the other two springs, where the Ca concentration was low, and the accumulated Li content was high. Ca and Li uptake is influenced by the initial metal concentrations from the growth medium. As observed in SW2 and SW3, the Li uptake by *S. natans* increased due to the availability of a high Li and low Ca concentration. The K content also decreased after exposure to Li, probably because Li shares the K transport carrier [56]. Hawrylak-Nowak et al. reported an increased K uptake when sunflower plants were exposed to a concentration of 50 mg-Li/L and no differences in K uptake when maize plants were exposed to Li concentrations between 0 to 50 mg-Li/L [51]. In plants, Li interacts with Na, K, and enzymes that require Mg. Therefore, Li can bind into sites that are not occupied by Mg [38].

The metal uptake in *S. natans* occurs in two stages: a fast stage, characterized by a rapid uptake, which can include physical processes (adsorption, ionic exchange, and chelation), and a slow stage, which includes biological processes, such as intracellular uptake, where the metal is transported through plasmalemma into cells [57]. Generally, K has a major role in transporting water and nutrients through the xylem. Thus, when low K contents are found in plants, the translocation of nitrates, phosphates, Ca, Mg, and amino acids decrease [58]. These findings agree with the current results, as the SM20 plants showed lower K and Mg contents, indicating the disturbing effect of Li and other elements on the nutrient transport system. Shahzad et al. reported that Li may replace Mg and Ca in plants during normal metabolic processes [32]. The slight decrease in Ca content indicated the competitive interactions between Ca and Li, as they share binding sites [59].

Exposure to Li led to various changes in the trace element content of *S. natans*. By increasing the Li content in the growth medium, the Fe content decreased from 1088 mg/kg DW in the control plant to 253 mg/kg DW in S20 and 274 mg/kg DW in S50. The decrease in nutrient uptake could be attributed to the changes induced by the high Li concentration in the functioning of essential element transporters [60]. In the case of simultaneous exposure to Li and Cu, Zn, and Cd, *S. natans* accumulated higher contents of Cu and Cd and lower amounts of Zn (Figure 2b). Following the exposure of *S. natans* to the Hoagland solutions enriched with 20 mg-Li/L, 20 mg-Cu/L, 20 mg-Zn/L, and 20 mg-Cd/L initial concentrations (SM20), the Cu, Zn, and Cd contents increased. However, the Li content was lower than in the plants exposed to 20 mg-Li/L (S20). In the Banpotoc, Borcut, and Botiza springs, the initial concentrations of Cu (0.21–3.81 µg-Cu/L) and Zn (2.56–11.7 µg-Zn/L) were much lower than in SM20. The plants exposed to the springs showed lower Cu and Zn contents than the control plant (SC), except for SW2, which showed a 2.8- and 2.4-fold greater Cu and a 2.7- and 2.0-fold greater Zn content. The highest Na, Mn, Fe, Cu, Zn, and Sr trace element contents were obtained in SW2, out of which the Na content (7.11 g/kg DW) was 1.9-fold higher than in the SC. The variation in metal accumulation could be explained based on a differential affinity towards metal and a competition between metal ions during the uptake [17].

Previous studies also reported the potential of *S. natans* to accumulate high levels of metals from water, though the capacity for metal accumulation varies for each treatment. Dhir et al. [61] observed a higher accumulation rate for Cr, Fe, Ni, Cu, Pb, and Cd than for Co, Zn, and Mn. Polechońska et al. reported *S. natans* as Cu, Fe, Ni, and Zn accumulator and Mn hyperaccumulator, while Reeves and Baker as Cd, Ni, Cu, Co, Pb, Cr, Zn, and Mn hyperaccumulator [16,62]. *S. natans* showed a considerable capacity to accumulate metals (Fe, Co, Cr, Pb, Cu, Zn, Cd, and Ni) by increasing the initial concentrations from 30 to 50 mg/L, but the tolerance index decreased, suggesting higher toxicity [17]. The variation in the metal absorption intensity by plants due to the synergistic or antagonistic effects between metals was previously reported in the literature [63]. Cu is an essential element of plants, acting as a cofactor for the enzymes involved in respiration and photosynthesis. However, in high concentrations, Cu is toxic. Therefore, plants regulate their Cu uptake through the metallochaperones (Cu-binding proteins) that deliver it to the plant cells [64]. Zn is a key element in the functioning of the enzymes involved in plant growth and development, such as polyphenol oxidase, ascorbic acid oxidase, and cytochrome oxidase [64,65]. Cu and Zn uptake may be cooperative and could be transported together in the plant cell via similar transporters and channels [64]. The higher uptake of essential elements, such as Zn and Cu, could be explained by their involvement in the enzymatic activity of plants, which may control the absorption intensity of toxic elements. For example, Zn may alleviate the toxic effects of Cd and Pb and, thus, enhance their uptake [66]. The similar properties of Zn and Cd also contribute to an increase in Cd uptake by using the same uptake mechanisms as Zn. The reduction of Mn and Cu uptake in the presence of Zn and Cd was reported for *Melissa officinalis.* This reduction could be attributed to the antagonistic effects between elements that use the same transporters [63,67].

In the present study, the influence of both essential and trace elements on Li uptake was observed. The accumulation of high concentrations of essential (Cu, Zn) and toxic (Cd) elements have antagonistic effects on the Li uptake in *S. natans*. As the Li-specific binding sites seem to lack in *S. natans,* the competition between the biologically active ions and Li uptake is regulated through highly selective channels, as well as active ion transporters. The influence of essential and toxic elements on Li uptake is not fully understood and deserves more research.

### 3.3. Bioaccumulation Factor

**Figure 3:**
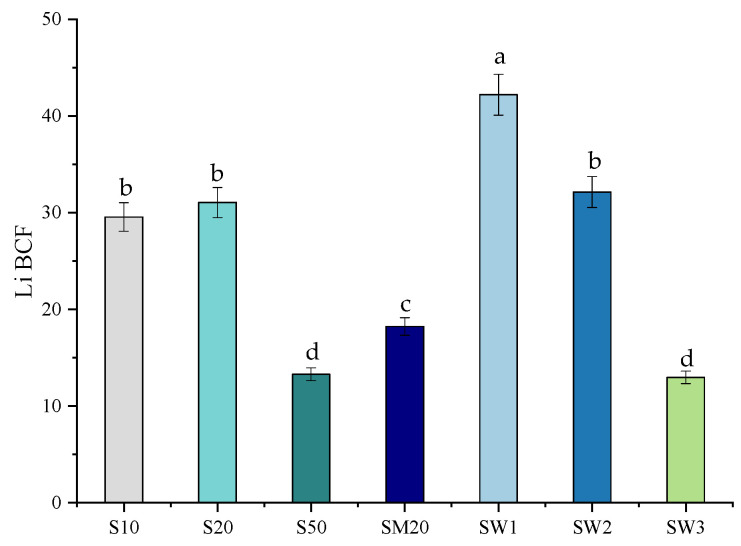
Bioaccumulation factor (BCF) of Li in *S. natans* plants exposed to Hoagland solutions enriched with 10 mg-Li/L (S10), 20 mg-Li/L (S20), 50 mg-Li/L (S50), 20 mg-Li/L, 20 mg-Cu/L, 20 mg-Zn/L, and 20 mg-Cd/L (SM20), and to Banpotoc (SW1), Borcut (SW2) and Botiza (SW3) spring waters. Bioaccumulation factors marked with different letters are significantly different at the *p* < 0.05 significance level.

Trace element accumulation (Cr, Co, Zn, Fe, Ni, Cd, Zn, and Mn) by *S. natans* was previously investigated [26]. Comparatively, the absorption of Li gained little attention. The bioaccumulation factor is an important index used to show the ability of a plant to accumulate a pollutant in its tissues [68]. The highest BCF value (Figure 3) was found in SW1 (42.2). Comparable BCF values were also obtained for SW2 (32.1), S20 (31.0), and S10 (29.5), while for S50, SM20, and SW3, the BCF decreased to half (13–18.2). This fact indicates that bioaccumulation is higher in the case of plants exposed to low Li contents than to high Li contents. BCF values, ranging from 1.16 to 1.96, were reported for *Apocynum pictum* grown in soils contaminated with 50–400 mg/kg Li [54]. High Li BCF values were also observed in *Lolium* sp., cultivated in natural conditions, where the Li content in the groundwater and soil samples ranged from 2.2 to 5.6 μg/L and 9.95 to 10 mg/kg, respectively [37].

### 3.4. Photosynthetic Pigments and Antioxidant Capacity of S. natans after Li Stress Conditions

**Figure 4:**
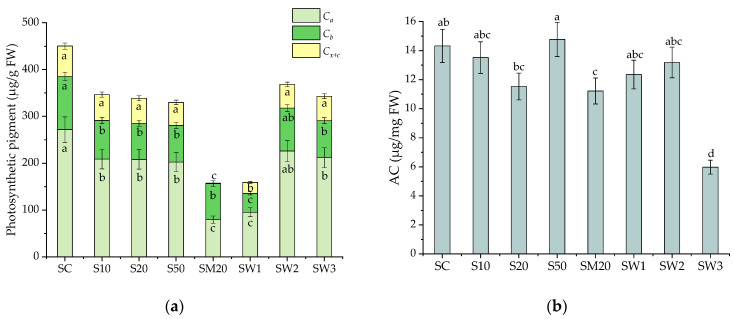
(**a**) Chlorophyll a (*C_a_*), chlorophyll b (*C_b_*), and carotenoids (*C_x+c_*) photosynthetic pigments concentration and (**b**) antioxidant capacity (AC) in *S. natans* grown in control (SC), and Hoagland solution enriched with 10 mg-Li/L (S10), 20 mg-Li/L (S20), 50 mg-Li/L (S50), 20 mg-Li/L, 20 mg-Cu/L, 20 mg-Zn/L, and 20 mg-Cd/L (SM20), and to Banpotoc (SW1), Borcut (SW2) and Botiza (SW3) spring waters. Photosynthetic pigment contents and antioxidant capacity marked with different letters are significantly different at the *p* < 0.05 significance level.

Exposure to Li led to changes in the photosynthetic pigment content and antioxidant capacity (AC, expressed as µg/mg FW) of *S. natans* (Figure 4). The *C_a_* slowly decreased from 271.8 µg/g FW in the SC to 208.6 and 208.1 µg/g FW in S10 and S20, respectively. The effect of Li on the *C_a_* content can be explained by the interaction between Li and Mg, which have similar physicochemical properties [69]. The *C_a_* concentration in S50 (202.3 µg/g FW) was similar to that of S10 and S20. The lowest value of the *C_a_* concentration was observed in SM20 (79.1 µg/g FW), probably due to the additional stress effects of Cu, Zn, and Cd. Li also had a negative effect on the *C_b_* content in S10, S20, and S50, as well as in SM20. The *C_x+c_* concentration slowly decreased with an increase in the Li concentration from S10 to S50. The lowest carotenoid concentration was obtained in the SM20 sample (1.1 µg/g FW).

Noticeably, the concentrations of the photosynthetic pigments in SW1, SW2, and SW3 were lower than the values obtained for the control sample. The *C_a_* concentration decreased from 271.8 µg/g FW in the control sample to 226.1, 211.8, and 95.1 µg/g FW in SW2, SW3, and SW1. The carotenoid concentration was low in SW1 (23.5 µg/g FW), while in SW2 (50.5 µg/g FW) and SW3 (52.2 µg/g FW), it was close to the control sample (65.0 µg/g FW). Generally, in stress conditions, the carotenoid content increases, but under prolonged stress, their content decreases, probably due to damage to the carotenoid biosynthetic pathway [69]. According to the carotenoid content, the W1 spring negatively affected the aquatic plants due to higher Ca, Fe, and Ba concentrations than the W2 and W3. The decrease in the chlorophyll content in the *Apocynum venetum* L. and *Brassica carinata* seedling plants exposed to Li was also reported [70,71]. Aside from Li, other essential and non-essential elements can affect the chlorophyll levels of aquatic plants. A previous study reported decreased chlorophyll and increased carotenoid contents in *Salvinia biloba* after a short time (48 h) exposure to 50 and 100 μM of Cu and Pb. In the same study, exposure to Cd and Zn did not reduce the chlorophyll content [72]. The increase in the carotenoids was attributed to the plant’s response to the induced stress through the quench of excess energy protecting the chlorophyll molecules from oxidative damage [72]. In the present study, in SW1, the chlorophyll and carotenoid levels decreased more visibly compared to SW2 and SW3. A possible explanation for this sharp decrease could be that *S. natans* was exposed to a low Li content and high Ca and Ba contents. The reduction in the plant’s photosynthetic activity following exposure to high Ba contents was also reported by Dridi et al. [73]. Similarly, a decrease in the photosynthetic pigment level in sunflower (*Helianthus annuus*) and soybean (*Glycine max*) exposed to Ba was reported [74,75]. Soybeans exposed to Ba also showed reduced photosynthetic activity and inhibited K uptake [74]. Therefore, more studies need to be conducted to clarify the competition between Ca, Ba, and Li, and their inhibition effect on the photosynthetic pigment levels.

The alterations in the photosynthetic pigment contents could be due to: (i) the low efficiency of the enzymes involved in chlorophyll biosynthesis; (ii) a decrease in the availability of Fe and Mn ions by the inhibition of their uptake in the presence of heavy metals; (iii) the peroxidation of chloroplast membranes during heavy metal stress; (iv) the formation of metal-substituted chlorophylls [61].

No notable differences were observed in the AC between the control (14.3 µg/mg FW) and S10–S50 samples (11.2–14.8 µg/mg FW). In contrast, a notable decrease in the AC was observed in the plants exposed to W3 (2.4-fold smaller than the SC value of 5.98 µg/mg FW). Previously, Upadhyay et al. demonstrated the negative effects of Cu, Zn, and Cd on the AC of *Potamogeton pectinatus* L. and *Potamogeton crispus* L. aquatic plants [76]. According to the obtained results, *S. natans* exposed to the enriched Hoagland solutions and spring waters exhibited small changes in the AC, suggesting the important role of the AC in the abatement of multi-element stress by scavenging free radicals and, hence, protecting the cells from oxidative damage [8]. The negative effect of Li on the AC of *S. natans* was not observed.

### 3.5. Elemental Composition

**Figure 5:**
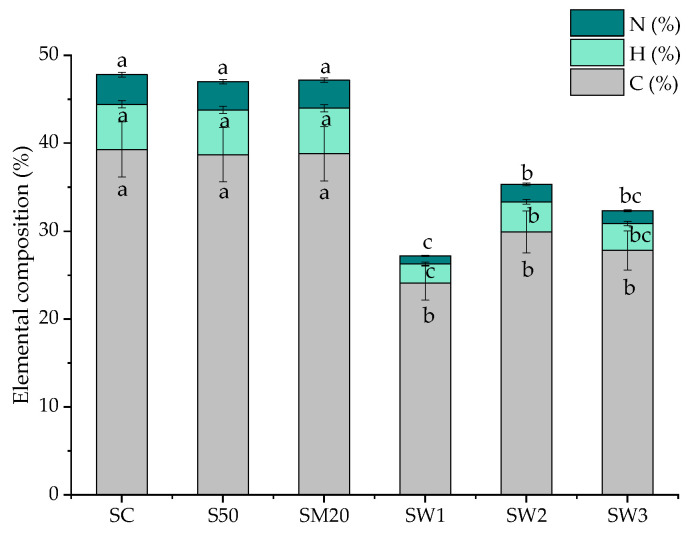
N, C, and H (%) contents in *S. natans* grown in control (SC) and Hoagland solution enriched with 10 mg-Li/L (S10), 20 mg-Li/L (S20), 50 mg-Li/L (S50), 20 mg-Li/L, 20 mg-Cu/L, 20 mg-Zn/L, and 20 mg-Cd/L (SM20), and to Banpotoc (SW1), Borcut (SW2) and Botiza (SW3) spring waters. N, C, and H contents marked with different letters are significantly different at the *p* < 0.05 significance level.

The changes in the elemental composition of *S. natans* exposed to different enriched Hoagland solutions and spring waters are shown in Figure 5.

The C, N, and H (%) contents remained unaffected in plants grown on S50 and SM20 compared to the control plants, although a decrease was observed in *S. natans* exposed to spring waters. The S content was below 0.01% in all the studied samples.

Changes in the elemental composition could be attributed to a photosynthetic efficiency reduction that determines an increase in non-photochemical quenching or damage of the reaction center molecules [17] and a decrease in biomass production. The differences observed in the elemental composition of *S. natans* plants exposed to the Hoagland solutions and spring waters could be due to a high amount of non-essential metal ions influencing metal uptake [77].

### 3.6. FT–IR Spectra

**Figure 6:**
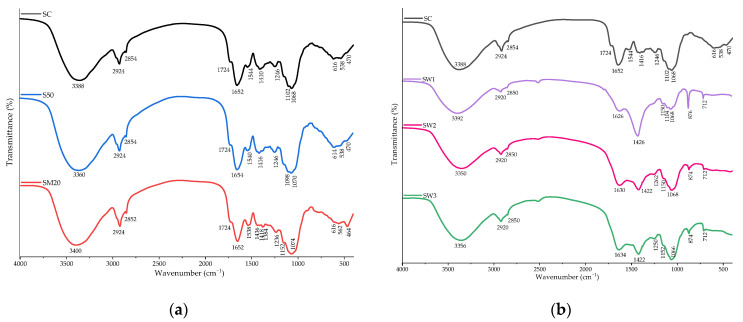
FT–IR spectra of *S. natans* grown in control (SC) and Hoagland solution enriched with 50 mg-Li/L (S50), 20 mg-Li/L, 20 mg-Cu/L, 20 mg-Zn/L, and 20 mg-Cd/L (SM20) (**a**) and Banpotoc (SW1), Borcut (SW2) and Botiza (SW3) spring waters (**b**).

The FT–IR spectra of *S. natans* exposed to Li are presented in Figure 6. The broad band at 3500–3000 cm^−1^ was attributed to the O–H and N–H stretching vibrations [78,79], while the intense peak, around 2920 cm^−1^, and the shoulder, around 2850 cm^−1^, are specific to the CH_3_ and CH_2_ stretching vibrations [78]. The peaks in the 1800–1200 cm^−1^ region are specific to the C=O stretching vibration, indicating the presence of ester-containing compounds commonly found in membrane lipid and cell wall pectin [78].

The peaks at 1654–1652 cm^−1^, 1580–1510 cm^−1^, and 1400–1200 cm^−1^ may also be attributed to amides [78,80,81]. The increase in these peaks could be an indication of the plant’s response to stress conditions, as amides are known to have an important role in the plant’s defense mechanism [82]. The peak at 1426 cm^−1^ was assigned to the C–H groups [83]. The peaks around 1250 cm^−1^ correspond to the C–O stretch in carboxylic acids, while those at 1130–1000 cm^−1^ correspond to the vibration of C–O–C and O–H in polysaccharides [84]. The peak around 1067 cm^−1^, observed in the SC, SW1, SW2, and SW3, can be assigned to the C–O stretching of alcohols and carboxylic acids [83]. In the *S. natans* grown in spring waters, a strong peak appeared around 880 cm^−1^, indicating the linkage of β–glycosides in the molecular structure of polysaccharides [79]. The bands below 800 cm^−1^ are the fingerprint region of the phosphate and sulphur functional groups [83,84].

The FT–IR spectra of S50 were similar to the control, suggesting that the plant that was exposed to Li did not determine the changes in the specific bands. At the simultaneous exposure to Li and Cu, Zn, and Cd, a supplementary band appeared at 1384 cm^−1^, being assigned to the amides. A peak at 1102 cm^−1^, attributed to the presence of C–O–C and O–H in the polysaccharides, disappeared, while the intensity of the peak at 464 cm^−1^, attributed to the phosphate and sulphate functional groups increased, indicating a plant response to the presence of environmental stress and the interactions between Li and the phosphate and sulphate functional groups.

## 4. Conclusions

*S. natans* is able to take up and accumulate Li from the nutrient solutions containing low or high Li concentrations and from spring waters. Exposure to a high Li content decreased K and Fe; however, it did not change the Na, Ca, and Mg concentrations. The Li uptake increased with the increase in the Li concentration in the growth medium, up to 20 mg-Li/L, and remained more or less constant at higher concentrations. The accumulation of Li was about two times lower in the case of simultaneous exposure to Li and Cu, Zn, and Cd than in the case of Li exposure. The Li bioaccumulation factor was higher for the plants exposed to a low Li content than for those exposed to high Li contents. Exposure to Li led to a decrease in antioxidant activity and photosynthetic pigments. The photosynthetic pigment content reflected the induced stress effect; therefore, the most affected plants were those simultaneously exposed to Li, Cu, Zn, and Cd.

## Figures and Tables

**Table 1:** Major (Na, Mg, K, and Ca) and trace (Li, Mn, Fe, Ni, Cu, Zn, Sr, and Ba) elements concentrations expressed as average ± standard deviation (*n* = 3) in Banpotoc (W1), Borcut (W2) and Botiza (W3) spring waters.

		W1	W2	W3
Major elements (mg/L)	Na	20.2 ± 1.6	484 ± 24	599 ± 30
	Mg	44.8 ± 3.6	52.8 ± 4.2	44.5 ± 3.6
	K	2.54 ± 0.20	7.18 ± 0.36	24.5 ±1.2
	Ca	386 ± 19	126 ± 10	154 ± 12
Trace elements (µg/L)	Li	68.6 ± 3.4	233 ± 12	2090 ± 104
	Mn	104 ± 5	351 ± 18	700 ± 35
	Fe	176 ± 9	35.3 ± 1.8	42.3 ± 2.1
	Ni	15.0 ± 0.7	11.0 ± 0.6	14.3 ± 0.7
	Cu	<0.21	3.49 ± 0.28	3.81 ± 0.72
	Zn	5.04 ± 0.46	11.7 ± 1.1	2.56 ± 0.23
	Sr	658 ± 33	1110 ± 56	1350 ± 67
	Ba	1010 ±51	46.6 ± 22.3	301 ± 15

## Data Availability

Not applicable.
